# Physical workplace violence in the health sector in Saudi Arabia

**DOI:** 10.1097/MD.0000000000034094

**Published:** 2023-07-21

**Authors:** Aseel Khaled Alhassan, Reem Tarik Alsaqat, Fahad Saleh Al Sweleh

**Affiliations:** a Department of Dentistry, King Khalid Hospital in AlKharj, Ministry of Health, Alkharj, Saudi Arabia; b Department of Dentistry, Restorative Division, Princes Noura University, Riyadh, Saudi Arabia; c Dental Clinic, King Saud University, Riyadh, Saudi Arabia.

**Keywords:** healthcare workers, physical violence, Saudi Arabia, workplace

## Abstract

Physical workplace violence (WPV) occurs worldwide, causing psychological and physical injuries. However, reports from Saudi Arabia investigating which specialty is the most exposed are scarce. This study aimed to determine the prevalence and circumstances related to physical WPV among all healthcare providers in Saudi Arabia in 12 months, as well as the consequences for both attackers and targets of physical WPV. This cross-sectional study included all healthcare providers registered with the Saudi Commission for Health Specialties who had worked for more than 1 year in the health sector in Saudi Arabia until May 2019. Researchers distributed the questionnaire to the participants via email. Descriptive statistics were used to describe the basic features of the data. Correlations between the categorically measured variables were explored using a chi-square test of independence. Overall, 7398 healthcare workers (HCWs) voluntarily participated in the study, 51.3% being men and 48.7% being women. The mean age was 40 ± 8.62 years), and most participants were of non-Saudi origin. Overall, 9.3% HCWs had encountered physical violence. Male HCWs, pharmacists, nurses, and HCWs of non-Saudi origin were significantly more exposed to physical violence. Furthermore, those with direct physical contact with patients and those working with male patients only were more exposed to physical violence. Physical WPV is an important issue faced by HCWs, particularly those who work night shifts or have direct contact with patients. Results showed that more support, specific strategies and policies to reduce violence occurrence, and protection for healthcare providers are required.

## 1. Introduction

The World Health Organization (WHO) defines violence as “the intentional use of physical force or power, threatened or actual, against oneself, another person, or against a group or community, that either results in or has a high likelihood of resulting in injury, death, psychological harm, maldevelopment, or deprivation.”^[1]^ Specifically, physical violence has been a well-known topic of research for many decades; however, it has received more attention recently. Physical violence is explicitly defined as “the use of physical force against another person or group that results in physical, sexual, or psychological harm. Physical violence includes beating, kicking, slapping, stabbing, shooting, pushing, biting, and pinching, among others.”^[2]^ Physical workplace violence (WPV) occurs less frequently than psychological WPV; however, it is not less important. Babiarczyk et al^[3]^ determined that 20% of nurses from 5 European countries had been exposed to physical WPV compared with 54% who experienced psychological WPV. In previous studies, the rate of physical WPV was 12.6% in China,^[4]^ 18.3% in Jordan,^[5]^ and 36.8% in Ethiopia.^[6]^ Locally, the percentage of physical WPV ranged from 5% to 67.6%; these vast differences between the findings could be attributed to the focus of those studies on a single city, some hospitals, and specialties or departments with higher risks to encounter violence.^[7–12]^ Although limited studies were conducted recently in Kingdom of Saudi Arabia, none was found to explain violence to healthcare workers (HCWs) in various circumstances, combine all workers in different settings, and explore some terminal cities regarding the exposure of their HCWs to violence.^[7–14]^ Physical attacks can cause psychological and bodily injuries to the victims that may range from minor to serious harm, leading to reduced workdays.^[15–17]^ Recently, WPV has gained attention; however, the low reporting rate remains a problem.^[18–20]^ There is also insufficient research encompassing all specialties and countries.^[21–23]^ Globally, multiple organizations, such as the WHO, have given considerable attention to this topic. Case studies conducted in different countries, such as Brazil, Bulgaria, and Lebanon,^[24]^ provide framework guidelines in the health sector to help workers prevent, work with, manage, and support people who have experienced WPV.^[25]^ The Occupational Safety and Health Administration provides information regarding the risk factors and strategies for preventing WPV, in addition to online training materials.^[26]^ The National Institute for Occupational Safety and Health provides online prevention courses and resources on this subject.^[27]^ The cause of physical violence varies worldwide. For instance, in poorly equipped hospitals, an increase in the demand will always exceed the supply within the public health system, which increases the risk of physical attacks, low reporting rates of incidents that need law enforcement interference, and stressful environments that cause anxiety in workers and make them more prone to physical violence.^[28,29]^ Nonetheless, working in some settings (e.g., in emergency departments and with patients with psychological problems) increases the prevalence of WPV.^[23]^

Although previous studies have focused on WPV, to the best of our knowledge, none have covered the whole of Saudi Arabia and all specialties. Additionally, few studies have focused on the association between WPV and independent risk factors, such as hospital characteristics (i.e., clinical role and rank).

Therefore, this study aimed to determine the prevalence of physical WPV over 12 months, the circumstances related to the event, and the consequences for the attacker and target person at all healthcare provider facilities in Saudi Arabia and to identify which group of healthcare providers is most susceptible.

## 2. Methods

### 2.1. Data collection

This study included all healthcare providers who are registered with the Saudi Commission for Health Specialties (SCFHS) and have been working for more than 1 year in the health sector (governmental or private) in Saudi Arabia by May 2019. The exclusion criteria were students, interns, employees of the administrative department, and providers who were not registered in SCFHS or had less than 1 year of work experience. Moreover, a convenient sampling technique was used where all eligible participants (i.e., physicians, pharmacists, nurses, midwives and health specialists, healthcare technicians, and technicians) were invited to participate in the study. Overall, 304,002 healthcare providers met the eligibility criteria.

The data were collected using a modified self-administered questionnaire developed by the Joint Program on Workplace Violence in the Health Sectors of the WHO, International Labor Organization, International Council of Nurses, and Public Services International. The questionnaire was translated into Arabic for staff who were not fluent in English. The questions that did not apply to Saudi Arabia were omitted.

A pilot test was conducted by distributing the questionnaire to 5 physicians, 5 dentists, 5 nurses, and 5 pharmacists who were Arabic and English speakers and had the clinical experience to validate the Arabic translation to avoid misunderstandings; these practitioners were excluded from the main study.

The questionnaire included questions related to the respondents’ demographic data, workplace characteristics, the experience of violent events during the previous 12 months, risk factors contributing to WPV, personal opinions, perceptions, attitudes, experiences, and participants’ knowledge of WPV. The researchers distributed the questionnaire by emailing it to the study population. To increase the response rate, the researchers sent reminder emails to the participants after 2 weeks.

### 2.2. Statistical analysis

Data analysis was performed using the SPSS (version 22; IBM, Armonk, NY). Descriptive statistics (frequency and table) were used to describe the basic features of the data. Continuous variables were expressed as mean and standard deviation, whereas categorical variables were expressed as frequencies and percentages. The Kolmogorov–Smirnov statistical test of normality and histograms were used to assess the statistical normality assumption of metric variables. The statistical homogeneity of variance assumption was evaluated using Levene’s test of homogeneity of variance. The chi-square test of independence was used to explore the correlations between the categorical variables. An independent samples *t* test was used to assess the mean differences of continuous variables across the levels of categorically binary measured variables.

A multivariate binary logistic regression analysis was conducted to assess the combined and individual associations between the relevant predictors of the exposure of HCWs to recent physical violence at the workplace. The association between the measured predictor variables and their outcomes was expressed as an odds ratio with a 95% confidence interval. Statistical significance was set at *P* < .05.

### 2.3. Ethical approval

This study was conducted following the guidelines of the Declaration of Helsinki. Approval was obtained from the institutional review board of King Saud University College of Medicine (approval number: E-18-3391) before the study was started. Written informed consent for participation, publication, and confidentiality was obtained from the study participants at the beginning of the survey.

## 3. Results

### 3.1. Demographic characteristics

Overall, 304,002 HCWs were recruited from the SCFHS database, and 7398 responded to the questionnaire. Among them, 51.3% and 48.7% were men and women, respectively. The participants’ mean age was 40 ± 8.62 years; and 60% were of non-Saudi origin. Nurses, midwives, and health specialists accounted for 38.1% of the study population, followed by physicians at 30.91%, healthcare technicians and ambulance technicians at 25.54%, and pharmacists at 5.43%. Most participants were employed full-time (89.86%) in the public or governmental sectors (72.47%) (Table 1).

**Table 1:** Descriptive analysis of healthcare worker’s sociodemographic and professional characteristics (N = 7398).

Characteristics	n (%)
Sex	
Male	3792 (51.3)
Female	3606 (48.7)
Age (yr)	
20–29	402 (5.4)
30–39	3752 (50.7)
40–49	2143 (29)
50–59	882 (11.9)
≥60	219 (3)
Nationality	
Saudi	2957 (40)
Non-Saudi	4441 (60)
Clinical role	
Physicians	2287 (40)
Pharmacists	402 (5.4)
Nurses, midwives, and health specialist	2819 (38.1)
Healthcare technicians and ambulance	1890 (25.5)
Rank/seniority	
Junior	4605 (62.2)
Senior	1876 (25.4)
Consultant	917 (12.4)
Experience years	
1–5	851 (11.5)
6–10	2334 (31.5)
11–15	1905 (25.8)
16–20	1025 (13.9)
≥21	1283 (17.3)
Working sector	
Semi-governmental organization	380 (5.1)
Private sector	1656 (22.4)
Public/governmental sector	5362 (72.5)
Employment type	
Full-time	7256 (98)
Part-time	78 (1.1)
Temporary/casual	64 (0.9)

### 3.2. Experience of WPV

Only 9.3% of HCWs experienced a physical attack at their workplace in the last 12 months, and most (94.9%) were instigated without weapons (Table 2). Patients attacked most HCWs (48.2%); 36.2% of HCWs were attacked by patients’ relatives. Moreover, most HCWs (60.6%) could not recollect the day on which the episodes of physical violence occurred; however, 8.2% of the recalled incidents occurred on Sundays. Furthermore, 37.4% of HCWs reported the incident to a senior staff member, and 33% told the offender to stop the violence (Tables 2 and 3).

**Table 2:** Healthcare workers’ perceptions and experience of physical workplace violence.

Variable	Total, n (%)
Occurrence of physical attack in the last 12 mo, n = 7398	
No	6713 (90.7)
Yes	685 (9.3)
Type of physical violence, n = 685	
Physical violence without a weapon	650 (94.9)
Physical violence with a weapon	35 (5.1)
Typical incidence of violence in your workplace, n = 685	
No	132 (19.3)
Yes	553 (80.7)
The attacked person, n = 685	
Patient/client	330 (48.2)
Relatives of patient/client	248 (36.2)
Other persons	37 (5.4)
Staff member	27 (3.9)
General public	18 (2.6)
Management/supervisor	16 (2.3)
External colleague/worker	9 (1.3)
Place of the incident, n = 685	
Inside the health institution or facility	654 (95.5)
At the patient’s/client’s home	18 (2.6)
Outside (on the way to work/health visit/home)	13 (1.9)
Time of the incident, n = 685	
07:00h–before 13:00h	204 (29.8)
13:00h–before 18:00h	131 (19.1)
18:00h–before 24:00h	180 (26.3)
24:00h–before 07:00h	56 (8.2)
Cannot remember	114 (16.6)

**Table 3:** Incidence of experience of physical workplace violence.

Response to the incident, n = 1373	
Reported it to a senior staff member	256 (37.4)
Told the offending person to stop	226 (33)
Tried to defend myself physically	148 (21.6)
Completed an incident/accident report form	146 (21.3)
Told a colleague	124 (18.1)
Took no action	107 (15.6)
Tried to pretend it never happened	104 (15.2)
Took another action	78 (11.4)
Told my friends/family members	60 (8.8)
Transferred to another position elsewhere	42 (6.1)
Pursued a prosecution against the offender	25 (3.6)
Sought counseling	24 (3.5)
Sought help from the medical association	23 (3.4)
Completed a compensation claim	6 (0.9)
Sought help from the Saudi Commission for healthcare workers	4 (0.6)
Preventability of the incident, n = 685	
No	182 (26.6)
Yes	503 (73.4)

### 3.3. Consequences of physical violence

As presented in Table 4, no injury was reported by 80.9% of HCWs because of the incident, with 21% of those who were physically offended requesting an action to be taken to explore the cause of the attack; however, 60.1% of HCWs suggested that no further investigation measures be taken. The management was the top entity that acted according to 81.5% of HCWs who were physically offended; however, 26.9% of HCWs requested police involvement. Moreover, 35.6% of HCWs who were physically attacked at their workplace stated that their supervisors offered them counseling; most of these HCWs (84%) were allowed to speak about the accident and report it, with 37.5% of HCWs also receiving another form of support. The overall satisfaction with how the management handled the physical attack incident was rated as 2.02 of the 5 satisfaction points, which is near-dissatisfactory. HCWs indicated that their primary reason for not reporting the incident was the belief that reporting the physical attack was useless (Table 5).

**Table 4:** Consequences of the physical violence.

Variable	Total, n (%)
Injured as a result of the incident, n = 685	
No	554 (80.9)
Yes	131 (19.1)
Mental or emotional repercussions of attack, n = 685	
Repeated, disturbing memories, thoughts, or images of the attack – mean (SD) Likert rating	3.16 (1.34)
Avoiding thinking about or talking about the attack or avoiding having feelings related to it – mean (SD) Likert rating	3.12 (1.32)
Being “super-alert” or watchful and on guard – mean (SD) Likert rating	3.85 (1.22)
Feeling like everything you did was an effort – mean (SD) Likert rating	3.69 (1.20)
Investigating the causes of the incident, n = 685	
No	412 (60.1)
Yes	144 (21)
Don’t know	129 (18.8)
The person who took action, n = 158	
Management/employer	106 (81.5)
Police	35 (26.9)
Other	10 (7.7)
Saudi Commission for healthcare specialties	4 (3.1)
Community	2 (1.5)
Medical association	1 (0.8)
The consequences for the attacker, n = 144	
None	38 (26.4)
Verbal warning issued	30 (20.8)
Don’t know	29 (20.1)
Reported to police	26 (18.1)
Other	13 (9)
Medical care discontinued	4 (2.8)
Aggressor prosecuted	4 (2.8)
The offer from the employer or supervisor, n = 224	
Opportunity to speak about/report it	231 (84)
Other support	103 (37.5)
Counseling	98 (35.6)
Incident handling satisfaction, n = 627 Mean (SD) Likert rating, 1 = V. dissatisfied, 5 = V. satisfied	
Very dissatisfied	334 (48.8)
Dissatisfied	86 (12.6)
Neutral	113 (16.5)
Satisfied	50 (7.3)
Very satisfied	44 (6.4)

**Table 5:** Reasons for not reporting the physical violence.

Reason for not reporting the incident, n = 788	
I thought it was useless	334 (53.3)
I was afraid of negative consequences	168 (26.8)
Other (please specify)	114 (18.2)
I did not know to whom to report the incident	65 (10.4)
It was not important	59 (9.4)
I felt ashamed	40 (6.4)
I felt guilty	8 (1.3)

### 3.4. Witnesses of incidents of physical violence

Table 6 shows that 32.6% of HCWs witnessed at least 1 physical attack at their workplace. Among them, 50% reported these incidents to their superiors; however, 16.9% of them were penalized for reporting that their peer was subjected to physical attacks.

**Table 6:** Witness of incidents of physical violence.

Variable	Total, n (%)
Witnessed incidents of physical violence in the last 12 mo, n = 7398	
No	4988 (67.4)
Yes	2410 (32.6)
Occurrence of the incident in the last 12 mo, n = 2410	
Once	800 (33.2)
2–4 times	1146 (47.6)
5–10 times	199 (8.3)
Several times a month	185 (7.7)
About once a week	50 (2.1)
Daily	30 (1.2)
Reporting an incident of workplace violence in the last 12 mo (witnessed or experienced), n = 7398	
No	6188 (83.6)
Yes	1210 (16.4)
Discipline for reporting an incident of workplace violence, n = 7398	
No	7194 (97.2)
Yes	204 (2.8)

### 3.5. Experience of physical attacks and their sociodemographic and professional factors

Female HCWs were projected to be at significantly lower risk of physical attacks at the workplace within the past 12 months than male HCWs. However, no significant association was found between the nationality of HCWs and their experience of physical violence at the workplace (*P* = .124). Additionally, nurses, midwives, health specialists, and pharmacists were more likely to be at a significantly higher risk of being subjected to physical attacks than physicians and healthcare technicians in the last year (*P* < .001). Furthermore, the seniority level of HCWs showed no statistically significant association with exposure to physical abuse at the workplace in the last year; however, the junior staff was at a slightly higher risk of physical attacks than other HCWs (*P* = .053). Moreover, the working sector and employment type of HCWs did not significantly correlate with their recent exposure to a physical attack at the workplace (Table 7).

**Table 7:** Association between the healthcare workers’ experience of physical attacks at the workplace and their sociodemographic and professional factors.

	Physically attacked in your workplace, n (%)		Test statistic	*P* value
	No = 6713	Yes = 685		
Sex				
Male	3409 (50.8)	383 (55.9)	χ^2^ (1) = 6.55	.010
Female	3304 (49.2)	302 (44.1)		
Age (yr)				
20–29	344 (5.1)	58 (8.5)	χ^2^ (4) = 66.99	<.001
30–39	3331 (49.6)	421 (61.5)		
40–49	1991 (29.7)	152 (22.2)		
50–59	837 (12.5)	45 (6.6)		
≥60	210 (3.10)	9 (1.3)		
Nationality				
Saudi	2702 (40.3)	255 (37.2)	χ^2^ (1) = 2.37	.124
Non-Saudi	4011 (59.7)	430 (62.8)		
Clinical role				
Physicians	2118 (31.6)	169 (24.7)	χ^2^ (3) = 36.70	<.001
Pharmacists	338 (5)	64 (9.3)		
Nurses, midwives, and health specialists	2526 (37.6)	293 (42.8)		
Healthcare technicians and ambulance	1731 (25.8)	159 (23.2)		
Rank/seniority				
Junior	4151 (61.8)	454 (66.3)	χ^2^ (2) = 5.9	.053
Senior	1715 (25.5)	161 (23.5)		
Consultant	847 (12.6)	70 (10.2)		
Experience in years				
1–5	759 (11.3)	92 (13.4)	χ^2^ (4) = 42.9	<.001
6–10	2063 (30.7)	271 (39.6)		
11–15	1727 (25.7)	178 (26)		
16–20	961 (14.3)	64 (9.3)		
>20	1203 (17.9)	80 (11.7)		
Employment sector				
Other-semi-governmental/private organization	355 (5.3)	25 (3.6)	χ^2^ (2) = 4.20	.110
Private—for-profit sector	1490 (22.2)	166 (24.2)		
Public/governmental sector	4868 (72.5)	494 (72.1)		
Employment type				
Full-time	6581 (98)	67 (98.5)	χ^2^ (2) = 1.60	.449
Part-time	74 (1.1)	4 (0.6)		
Temporary/casual	58 (0.9)	6 (0.9)		

### 3.6. Working conditions and experience of physical attacks

Table 8 shows that HCWs working evening shifts and those with direct interaction and physical contact with patients were more likely to be at a significantly higher risk of recent physical violence at their workplaces (*P* < .001). Additionally, HCWs working with elderly patients, adults, adolescents, children, and infants were significantly more likely to be exposed to physical violence at the workplace (*P* < .001); however, HCWs working with newborns were not as likely to experience physical attacks. Moreover, the analysis showed that HCWs working with only male patients were more likely to be exposed to physical violence at the workplace (*P* < .001). The workers who spent more than 50% of their job working at hospitals were significantly more likely to have had recent physical violence in their work areas (*P <* .001); those spending more than 50% of their work in ambulance services and community-based jobs were significantly more likely to have had recent physical violence while working (*P =* .010).

**Table 8:** Association between healthcare workers’ experience of physical attacks at the workplace and their working conditions factors.

Variable	Physically attacked in your workplace (%), n = 7398		Test statistic	*P* value
	No = 6713	Yes = 685		
Work in shifts				
No	3003 (44.7)	178 (26)	χ^2^ (1) = 89.15	<.001
Yes	3710 (55.3)	507 (74)		
Working time between 18:00 (6 pm) and 07:00 (7 am)				
No	2582 (38.5)	139 (20.3)	χ^2^ (1) = 88.30	<.001
Yes	4131 (61.5)	546 (79.7)		
Interacting with patients/clients				
No	732 (10.9)	15 (2.2)	χ^2^ (1) = 51.99	<.001
Yes	5981 (89.1)	670 (97.8)		
Routine direct physical contact (washing, turning, lifting) with patients/clients				
No	3154 (47)	231 (33.7)	χ^2^ (2) = 137.99	<.001
Yes	2830 (42.2)	439 (64.1)		
Not applicable	729 (10.9)	15 (2.2)		
Patients/clients you most frequently work with are (tick all appropriate)				
Newborns	1323 (18.4)	138 (20.2)	χ^2^ (1) = 1.37	.242
Infants	1441 (21.5)	172 (25.1)	χ^2^ (1) = 4.84	.028
Children	2460 (36.6)	291 (42.5)	χ^2^ (1) = 9.10	.003
Adolescents	3116 (46.4)	431 (62.9)	χ^2^ (1) = 67.82	<.001
Adults	5219 (77.7)	620 (90.5)	χ^2^ (1) = 60.91	<.001
Elderly	3737 (55.7)	489 (71.4)	χ^2^ (1) = 62.70	<.001
Sex of the patients you most frequently work with				
Unspecified/NA	729 (10.9)	15 (2.2)	χ^2^ (3) = 73.64	<.001
Female	523 (7.8)	50 (7.3)		
Male	552 (8.2)	98 (14.3)		
Male and female	4909 (73.1)	522 (76.2)		
Where to spend most of the time (more than 50%) in the main job if not in the hospital, n = 1512				
Other	755 (54.6)	52 (48.6)	χ^2^ (6) = 14.52	.024
Ambulance	55(4)	11 (10.3)		
Rehabilitation center/convalescent home	44 (3.2)	6 (6.5)		
Health center	435 (31.4)	27 (25.2)		
Hospice	5 (0.4)	0		
Home for the elderly/nursing home	5 (0.4)	2 (1.9)		
Community service (e.g., home care, outreach service, health visiting)	85 (6.1)	9 (8.4)		

### 3.7. Experience of physical attacks and violence reporting characteristics of the hospital

HCWs exposed to physical violence had a significantly higher anxiety level after experiencing WPV (According to the Likert rating, the mean worry = 3.94, SD = 1.13) than those who had not been recently physically attacked at work (mean worry = 2.74, SD = 1.30; *P* < .001). Additionally, HCWs serving at a facility with in-place guidelines for reporting violence were significantly less exposed to physical violence than those working without violence reporting guidelines, *P* < .001 (Table 9). Furthermore, the analysis indicated that knowledge of how to use reporting guidelines did not significantly correlate with exposure to physical violence at the workplace (*P* = .058); however, receiving encouragement to report an incident from the administration of the facility resulted in significantly lower exposure to physical abuse among HCWs (*P* < .001). All sources of encouragement to report violence significantly reduced exposure to physical violence at the workplace (*P* < .001).

**Table 9:** Association between healthcare workers’ experience of physical attacks at the workplace and their hospital violence reporting guidelines characteristics.

Variable	Physically attacked in your workplace (%), n = 7398		Test statistic	*P* value
	No = 6713	Yes = 685		
Worried about violence in the current workplace	2.74 (1.30)	3.94 (1.13)	*t*(7396) = 23.50	<.001
Presence of procedures for reporting violence				
No	1840 (27.4)	256 (37.4)	χ^2^ (1) = 88.30	<.001
Yes	4873 (72.6)	429 (62.6)		
Knowing how to use a report				
No	733 (15)	50 (11.7)	χ^2^ (1) = 3.60	.058
Yes	4140 (85)	379 (88.3)		
Encouragement to report workplace violence				
No	2355 (35.1)	384 (56.1)	χ^2^(1) = 117.3	<.001
Yes	4358 (64.9)	301 (43.9)		
Person who encourages reporting				
Management/employer	3680 (54.8)	252 (36.8)	χ^2^ (1) = 81.15	<.001
Colleagues	1388 (20.7)	96 (14)	χ^2^ (1) = 17.2	<.001
Saudi commission for health specialist	537 (8)	33 (4.8)	χ^2^ (1) = 8.85	.003
Medical association	174 (2.6)	7 (1)	χ^2^ (1) = 6.42	.011
My own family/friends	355 (5.3)	21 (3.1)	χ^2^ (1) = 6.35	.012
Other persons	278 (4.1)	29 (4.2)	χ^2^ (1) = .01	.908

### 3.8. The influence of exposure to physical violence

**Figure 1.**
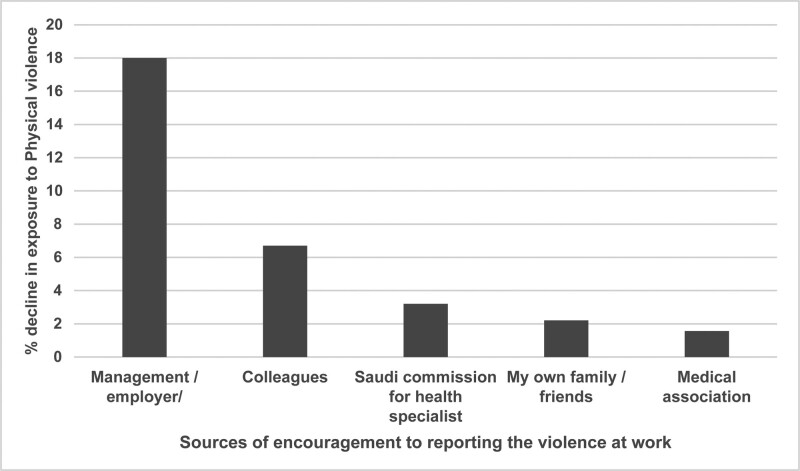
The percentage difference (i.e., decline) in exposure to physical violence, broken down by sources of violence reporting encouragement at the workplace.

Figure 1 shows the sources of encouragement for reporting violence. Encouragement from leaders and supervisors was associated with the greatest difference in reducing exposure to physical violence, followed by encouragement from colleagues, the SCFHS, family and friends, and the medical association.

### 3.9. Multivariate logistic binary regression analysis results

**Figure 2.**
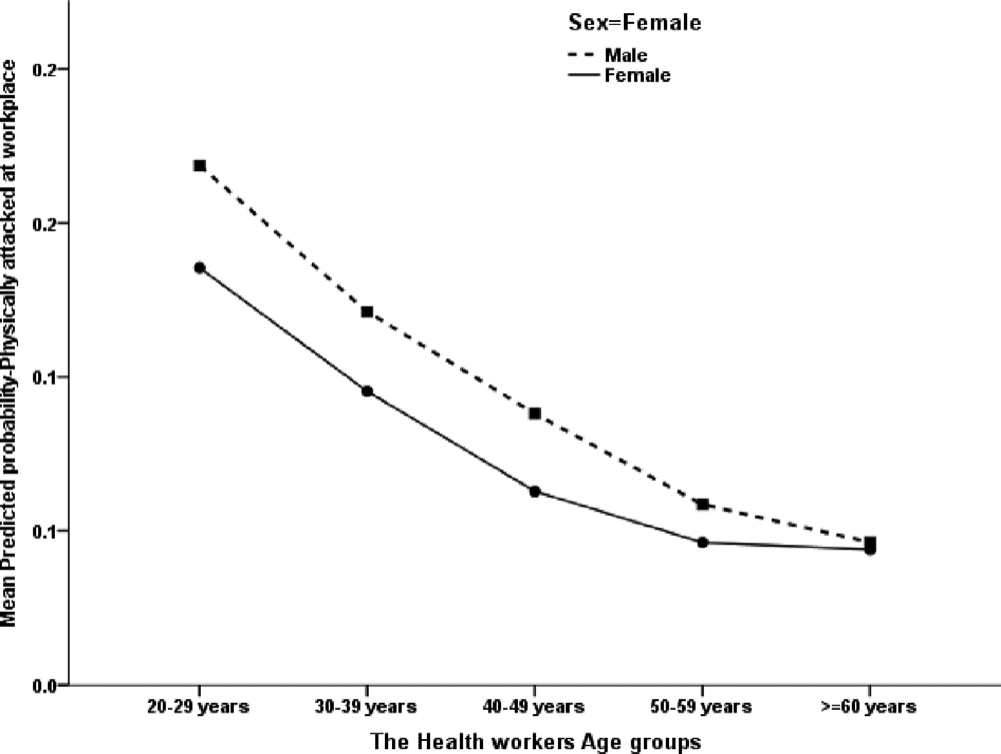
The association between the health workers’ age groups and sex with their mean predicted probability of being physically attacked at work.

As presented in Table 10, on average, female HCWs were projected to be at a significantly lower risk of experiencing a recent physical attack at the workplace within the past 12 months than male HCWs (*P* = .023). Additionally, HCWs aged 20 to 29 years old were significantly more likely (40.2% more likely) to have been physically abused at the workplace than those aged 30 years or older (*P* = .40) (Fig. 2). Non-Saudi HCWs were significantly more exposed (40.6% more exposed) to physical harassment than Saudi HCWs (*P* = .001). Furthermore, pharmacists were predicted to be at a significantly higher (3.02 times higher or 200.2% higher) risk of being physically attacked at the workplace than physicians (*P* < .001); however, nurses were predicted to be at a significantly higher risk (66.9% times more) of being physically attacked at the workplace than physicians (*P* < .001). Conversely, the analysis model indicated that the seniority level of HCWs did not correlate significantly with their exposure to recent physical abuse at the workplace (*P* = .263). The presence of violence reporting guidelines did not significantly correlate with the HCWs’ odds of being physically abused (*P* = .161), although such guidelines for managing violence predicted less (though not statistically significant) exposure to physical violence. However, working within an environment that encourages reporting violence anticipated significantly lower odds of physical abuse among HCWs. HCWs working in such environments were expected to be at a considerably lower (44% times lesser) risk of having been physically attacked recently (*P* < .001) than those working in environments that were not supportive/did not encourage reporting violence of any kind at the workplace, which accounted for the other predictors.

**Table 10:** Multivariate logistic binary regression analysis of the predictors of healthcare workers’ exposure to recent physical violence at the workplace (n = 7398).

	Multivariate adjusted OR	95% CI for OR		*P* value
		Lower	Upper	
Job = Pharmacist	3.024	2.055	4.451	<.001
Has direct physical contact with patients	1.918	1.568	2.346	<.001
Worry level from violence at work -mean score	1.906	1.769	2.054	<.001
Job = Nurse	1.669	1.261	2.208	<.001
Works with male patients only	1.527	1.159	2.013	.003
Works evening shifts (18:00–07:00)	1.512	1.224	1.868	<.001
Job = Technician	1.461	1.051	2.032	.024
Works with adults	1.456	1.060	1.999	.020
Works with adolescents	1.431	1.192	1.718	<.001
Nationality = non-Saudi	1.406	1.155	1.713	.001
Age: 20–29 yr	1.402	1.015	1.936	.040
Spends 50% or more of work time at the hospital	1.239	0.948	1.618	.116
Seniority level	1.100	0.931	1.299	.263
Presence of violence reporting guidelines at the workplace	0.865	0.707	1.059	.161
Sex = Female	0.787	0.640	0.967	.023
Workplace encouraging reporting of violence	0.560	0.458	0.684	<.001
Works with neither patient genders	0.220	0.111	0.434	<.001

CI = confidence interval, OR = odds ratio.

## 4. Discussion

WPV is a global problem that is also found in Saudi Arabia,^[30]^ with many articles^[31]^ assessing the prevalence of WPV in some Saudi Arabian cities and specialties.^[7–9,31]^ To the best of our knowledge, no studies have focused on HCWs from all cities in Saudi Arabia. This comprehensive study with many participants focused on physical violence against all healthcare providers across Saudi Arabia.

Few participants had experienced physical violence in the last 12 months. Comparatively, the extent of physical violence was less than that reported by Mohamed^[31]^ and more than that reported by Alharthy et al,^[7]^ Al-Turki et al,^[8]^ and El-Gilany et al^[9]^ However, the study by Mohamed^[31]^ only included nurses working in Riyadh City with most working in emergency and psychiatric units, thus the higher reporting rates. The other 3 local studies also focused on specific specialties, certain cities, or primary care centers.^[7–9]^

Internationally, this result is generally lower than that in Iran, Ethiopia, China, Italy, Palestine, and Turkey.^[15,32–36]^ The results from Egypt were consistent with this result, with the same percentage of incidence of physical abuse.^[37]^ Additionally, the result is consistent with the systematic review and meta-analysis conducted by Binmadi et al^[22]^ The variation between the countries may be due to the different perceptions of violence between different cultures, under-reporting, or due to some studies focusing on certain specialties that had a higher incidence of physical abuse.

Physical attacks usually originated from the patients and their relatives, similar to the findings of Mohamed,^[31]^ Fallahi-Khoshknab et al,^[32]^ Binmadi et al,^[22]^ and others.^[8,38]^ However, some studies, such as those conducted by Alharthy et al,^[7]^ El-Gilany et al,^[9]^ and Duan et al,^[39]^ reported the opposite; these studies determined that attacks originating from patients’ relatives were more frequent than physical attacks originating from the patients themselves. Approximately half of all HCWs registered in the SCFHS are of non-Saudi origin. Attacks originating from patients against non-Arabic-speaking HCWs could be due to a misunderstanding between the patient and healthcare provider, may not speak Arabic, or have a different language that could cause communication barriers.^[40,41]^ Consistent with the results of this study, those of previous studies revealed that most incidents occurred outside the institution^[7]^ and inside the institution.^[8,32,42]^

We found that HCWs working in the public and private sectors have the same risk of encountering physical violence. In contrast, governmental HCWs in Ethiopia had a higher risk of experiencing physical violence than those in the private sector.^[33]^ The lack of differences in our results may have been due to patients seeking treatment in both private and public hospitals in Saudi Arabia equally, as all hospitals are generally well-equipped and have well-trained staff, which increases healthcare provider loads at all times and, consequently, patient waiting times.

Nurses, midwives, and healthcare specialists (except pharmacists) are the top groups among all other professional groups to encounter physical violence. It was impossible to compare this study’s results to those of other studies because most studies did not include all HCW specialties; some studies categorized HCWs into physicians and nurses, whereas others investigated a single specialty. El-Gilany et al^[9]^ included more than 5 categories and determined that physicians rather than pharmacists were more prone to experience physical violence, whereas Al-turki et al^[8]^ found that clerks rather than pharmacists experienced physical violence more frequently. Nurses frequently experience WPV owing to their vital role in the hospital setting.^[43]^ Nurses are usually the first healthcare providers to interact with patients, patients’ family members, or caregivers. They also monitor signs and symptoms periodically, increasing their risk for WPV.^[33,43–46]^

This study showed that physical attacks usually occurred between 0700 and 1300 on the first day of the week (Sunday in Saudi Arabia). This finding was similar to those of Al-turki et al,^[8]^ El-Gilany et al,^[9]^ and Ferri et al,^[15]^ but differed from those of Abbas et al^[37]^ and Kitaneh et al,^[35]^ who reported that most attacks occurred during night shifts. In our study, most attacks may have occurred on the first day of the week as more people sought treatment after weekends, which may have led to overcrowding, increased waiting time, and increased workloads of HCWs. Conversely, most of the victims were working night shifts; this could be explained by the under-reporting of night attacks, as fewer staff are available to take time off for reporting.

The participants responded to the incident by reporting it to a senior staff member; some informed the attacker to stop, while others took no action. Very few pursued prosecution or completed a compensation claim. In a study by Alharthy et al,^[7]^ the participants usually asked the aggressors to stop, then attempted to physically defend themselves, while only a few took no action.

The participants in most studies stated that violence could be prevented.^[8,13,37]^ Availability of security personnel, liaison with police, and a penalty for perpetrators are the most frequent suggestions to prevent and control violence.^[9]^ Physical violence incidents caused injuries to some of the victims, and half of them consequently needed treatment, whereas others experienced psychological changes after the attack. Physical violence may lead to HCWs taking some time away, as over one-fifth of the participants took at least 1 day off from work after the incident. This may lead to a shortage of staff and financial loss for the institution.^[39,47]^ WPV negatively affects workers’ job satisfaction and leads to more job burnout.^[37,39]^ Work performance was diminished, and some participants felt sad, stressed, ashamed or guilty, bothered, suspicious, and angry after a WPV incident.^[8,9,35]^ WPV also causes psychological consequences,^[15]^ such as mental stress, which results in sleep disturbance and deteriorated health.^[34]^

A high proportion of physical violence incidents occurred with no action taken to investigate the causes of the events.^[13,32,42]^ In this study, only a few respondents reported an investigation, most of which were performed by the management or employer of the institution. This finding is similar to that of El-Gilany et al,^[9]^ who stated that most of the time, no action was taken; only 4 perpetrators were issued a verbal warning by the directors of primary health care centers.

A high percentage of victims did not report the incident, similar to previous studies’ findings,^[7,15,32,35,38]^ indicating that the rules or their implementation were inadequate or that there was a lack of knowledge regarding the rules. Another explanation for not reporting the attacks may be a lack of encouragement to report, having a previous fruitless experience, or fear of revenge from the attacker or their families.^[10]^ Gacki-Smith et al^[48]^ mentioned that having no physical injury as evidence of physical violence could prevent nurses from reporting the incident or that they wanted to protect their image by not giving the impression of being weak or unqualified. Lu et al^[29]^ reported that nurses would report more cases of WPV when there was a reporting system in the hospital, possibly because the abovementioned study was performed at psychiatric hospitals that had more violent events or because workers were more aware of when and how to report them because they knew the system.^[29]^ Some HCWs who reported the incidents were victimized or disciplined/penalized for reporting the incident, which highlighted a substantial amount of blame for reporting healthcare workplace physical abuse, which may be another contributing reason for not reporting any future accidents.

HCWs exposed to physical violence reported a significantly higher worry level from their WPV than those who had not recently been physically attacked at work.^[3,4]^ Additionally, HCWs serving at a facility with in-place guidelines for reporting violence were, on average, significantly less likely to be exposed to physical violence than those working in facilities without violence reporting guidelines. Though knowledge of how to use reporting guidelines did not significantly correlate with exposure to physical violence at work, encouragement to report violence by facility administration significantly reduced exposure to physical abuse among HCWs.

Therefore, further studies are needed, mainly of a longitudinal nature, to explore the reasons for physical violence and to implement solutions accordingly. Educational programs are required for HCWs, patients, and their relatives, and increasing awareness through the media is important. A more encouraging environment to report every violent incident with strict consequences for the attackers should be implemented. More importantly, new regulations (e.g., more staff members, shorter waiting times, and more support, such as prevention programs) are necessary. The main limitation of this study is its use of a retrospective self-report questionnaire, which might cause recall bias. Additionally, although this study had a sufficiently large number of participants to be considered a convenient sample, the results cannot be generalized to the population. The strength of this study involves all HCWs in both government and private institutions in Saudi Arabia, unlike previous studies that mainly focused on emergency departments or nurses.

In conclusion, the rate of physical WPV in Saudi Arabia is lower than what has been reported in previous literature in other countries; however, it remains a risk faced by HCWs, particularly those working night shifts and having direct contact with patients. The prevalence was the highest among pharmacists, nurses, midwives, and healthcare specialists, followed by healthcare technicians and physicians. This result shows that more support and specific strategies and policies to reduce the occurrence of WPV, protect healthcare providers, and prevent the event are required. Under-reporting the attacks may give an incorrect indication of the magnitude of the problem; therefore, more education and additional research in the Kingdom of Saudi Arabia are needed.

## Acknowledgments

A special thanks to the Saudi Commission for Health Specialties, who helped us reach the healthcare providers registered at their institution.

## Author contributions

**Conceptualization:** Fahad Saleh Al Sweleh.

**Data curation:** Aseel Khaled Alhassan, Reem Tarik Alsaqat, Fahad Saleh Al Sweleh.

**Formal analysis:** Fahad Saleh Al Sweleh.

**Investigation:** Aseel Khaled Alhassan, Reem Tarik Alsaqat.

**Methodology:** Aseel Khaled Alhassan, Reem Tarik Alsaqat, Fahad Saleh Al Sweleh.

**Project administration:** Aseel Khaled Alhassan.

**Resources:** Aseel Khaled Alhassan, Reem Tarik Alsaqat.

**Software:** Reem Tarik Alsaqat.

**Supervision:** Fahad Saleh Al Sweleh.

**Writing – original draft:** Aseel Khaled Alhassan, Reem Tarik Alsaqat.

**Writing – review & editing:** Aseel Khaled Alhassan, Fahad Saleh Al Sweleh.
